# Haloperidol Use in the Emergency Department for Gastrointestinal Symptoms: Nausea, Vomiting, and Abdominal Pain

**DOI:** 10.14309/ctg.0000000000000362

**Published:** 2021-06-01

**Authors:** Dariush Shahsavari, Kaitlin Reznick-Lipina, Zubair Malik, Mark Weiner, Asad Jehangir, Zachary D. Repanshek, Henry P. Parkman

**Affiliations:** 1 Department of Gastroenterology, Temple University, Philadelphia, Pennsylvania, USA;; 2 Lewis Katz School of Medicine at Temple University, Philadelphia, Pennsylvania, USA;; 3 Weill Cornell Medical College, New York, New York, USA;; 4 Department of Gastroenterology, Augusta University, Augusta, Georgia, USA;; 5 Department of Emergency Medicine, Temple University, Philadelphia, Pennsylvania, USA.

## Abstract

Haloperidol (HL) has successfully been used for nausea and abdominal pain in emergency departments (EDs). This study examines outcomes and predictive factors for clinical improvement of patients presenting to an ED with gastrointestinal (GI) symptoms (nausea, vomiting, and abdominal pain) who received HL.

**METHODS:**

Review of patients' records who presented to our ED between August 2016 and March 2019 with GI symptoms and received HL. *International Classification of Diseases, Tenth Revision* codes were used to identify patients.

**RESULTS:**

In all, 281 patients (410 encounters) presented to the ED with GI symptoms and received HL for their symptoms: 66% were women, 32% had diabetes, 68% used marijuana, and 27% used chronic opioids. Patients received HL 1.1 ± 0.3 times with dose 2.5 ± 3.0 mg, mostly intravenously (84.6%). Total ED length of stay was 7.5 ± 3.9 hours (3.2 ± 2.1 hours before HL and 4.4 ± 3.4 hours after). Approximately 4.4% of patients developed side effects to HL, including 2 patients with dystonia which improved with medication before discharge. Most patients (56.6%) were discharged home while 43.2% were admitted to hospital mostly because of refractory nausea or vomiting (70.1%). Receiving HL as the only medication in the ED led to lower hospital admission (odds ratio = 0.25, *P* < 0.05). Diabetes, cannabinoid use, anxiety, male sex, and longer ED stay were associated with increased hospital admissions.

**DISCUSSION:**

Most patients treated in our ED with HL for GI symptoms, particularly nausea, vomiting, and/or abdominal pain, were successfully treated and discharged home. HL use seemed relatively safe and, when used as the only medication, led to less frequent hospital admissions.

## INTRODUCTION

Gastrointestinal (GI) symptoms contribute substantially to health care use including emergency department (ED) visits and hospitalization in the United States (1). Abdominal pain is the symptom most responsible for ED visits, followed by nausea/vomiting. The utilization of ED services has dramatically increased over the past 2 decades (2). In the ED, various therapies have been used to treat nausea and vomiting especially from gastroparesis, including metoclopramide, ondansetron, lorazepam, and, most recently, haloperidol (HL).

HL, a butyrophenone, is a potent dopamine antagonist. It is a first-generation antipsychotic which is used in schizophrenia and related disorders (3). HL also acts on the gastric and cerebral chemoreceptor trigger zones reducing nausea and vomiting (4). It also has shown analgesic effects, the mechanism of which is not fully understood, although some literature report N-methyl-D-aspartate receptor modulation (5–7). HL has also been demonstrated to be efficacious in cyclic vomiting syndrome (CVS) and cannabinoid hyperemesis syndrome (CHS) (8,9). HL has successfully been used in the past as an antiemetic in general surgery and oncology (10–12) and more recently in EDs for diabetic gastroparesis (13). The Haloperidol Undermining Gastroparesis Symptoms trial demonstrated that HL in the ED can result in a reduction in morphine analgesia administered and a reduction in hospital admissions for patients presenting with an acute exacerbation of gastroparesis (14). This was further examined in a randomized, double-blind, placebo-controlled trial demonstrating a significant mean reduction in pain and nausea for gastroparesis patients receiving HL in the ED (15). Of note, these studies have been conducted in patient populations with specific conditions (e.g., cancer, gastroparesis, and cyclic vomiting).

Patients with gastroparesis and other disorders with nausea and vomiting such as CVS often have symptoms over years and cared for by gastroenterologists and primary care physicians. However, for acute exacerbations, these patients often present to EDs for symptomatic control. Our overall aim was to examine the use of HL in patients who present to the ED with GI symptoms. The primary aim was to characterize the use of HL at our tertiary care center's ED and to examine the outcomes of patients who present with nausea, vomiting, and/or abdominal pain from a variety of disorders including gastroparesis, CVS, and CHS who receive HL for treatment of their symptoms.

## METHODS

This was a retrospective review of patients' electronic medical records (EMRs) who presented to our ED at Temple University Hospital between August 2016 and March 2019 with GI symptoms (nausea, vomiting, and abdominal pain) and received HL. *International Classification of Diseases, Tenth Revision* codes were used to identify these patients: R11 nausea and vomiting, R11.0 nausea, R11.10 unspecified vomiting, R11.1 vomiting, R11.11 vomiting without nausea, R11.14 bilious vomiting, R11.2 unspecified nausea with vomiting, R10.9 unspecified abdominal pain, R10.13 epigastric pain, R10.81 abdominal tenderness, R10.819 abdominal tenderness with unspecified site, K31.84 gastroparesis, G43.A0 cyclical vomiting, G43.A1 intractable cyclical vomiting, and K30 functional dyspepsia.

Patients younger than 18 years and patients who left the ED before being seen by medical staff were excluded from this study. Electronic records including physician notes were examined to confirm coding was appropriate with the actual presenting symptoms and that HL was administered during their ED stay.

Recurrent presentations by the same patients were considered a separate encounter and were included when they were within the period mentioned above. A second data set was used for demographic information which only included unique patients to avoid duplication.

Demographic information such as age, sex, body mass index (BMI), ethnic/racial group, and presence of diabetes, as well as other information such as electrocardiogram, medication doses, chronic opioid (either prescription medications or illicit opioids), or marijuana use, was obtained from our EMR. In addition, Pennsylvania Prescription Drug Monitoring Program database was checked to capture all patients on chronic opioids.

The Spearman rho test was used to show correlation between continuous variables. Binary multiple logistic regression methods were used to make predictive models for admission to hospital, return to the ED within 30 days; outpatient follow-up as dependent variables (outcomes); age, sex, race, BMI, diabetes, underlying GI disorders, psychiatric disorders, and presenting symptoms; and other medications given in the ED as independent covariates (predictors).

During the same period, 753 patients were identified who presented to our ED with similar GI symptoms and did not receive HL. Of these patients, 280 patients with 410 ED encounters were randomly selected using SPSS (IBM, Armonk, NY) software randomizer as a control group for further multivariable logistic regression models. SPSS software version 23 was used for all analyses.

## RESULTS

From August 2016 to March 2019, there were 456,000 visits to the Temple University Hospital ED. There were 281 unique patients (410 total ED visits) with GI symptoms including nausea, vomiting, and abdominal pain and received HL for their symptoms. Demographic features are shown in Table 1. Approximately 65.8% of patients were women. The mean age was 37.3 ± 13.1 years (range 18–87 years), and average BMI was 27.1 ± 6.7. African Americans were the most common patients (52.3%), followed by Latinos (19.9%) and whites (16.8%). Approximately 32.4% had diabetes (24.6% insulin-dependent and 7.5% non–insulin-dependent), 68.3% used marijuana, and 26.6% were on chronic opioids.

**Table 1.** Demographics of patients presenting with GI symptoms who received HL

	N	Percentages
Total number of ED visits	410	
Total number of patients	281	
Sex		
Female	185	65.84
Male	96	34.16
Race		
African American	147	52.31
Latino	56	19.93
White	47	16.73
Other	31	11.03
Diabetes		
Nondiabetic	190	67.62
Diabetes		
Insulin-dependent diabetes	69	24.56
Non–insulin-dependent diabetes	21	7.47
Total	90	32.38
Mean age ± SD	37.27 ± 13.09	
Mean BMI ± SD	27.07 ± 6.68	
Top *ICD-10* diagnosis codes		
1: Nausea with vomiting, unspecified (R11.2)	125	31.46
2: Gastroparesis (K31.84)	70	17.07
3: Cyclical vomiting (G43.A0)	38	9.76
4: Vomiting, unspecified (R11.10)	32	7.80
5: Cyclical vomiting, intractable (G43.A1)	21	5.12
Cannabinoid	280	68.29
Chronic opioids	109	26.59
Underlying GI disorder		
Gastroparesis	163	39.76
CHS	116	28.29
CVS	43	10.49
PUD	24	5.85
Chronic abdominal pain	15	3.66
GERD	7	1.71
Psychiatric disorders		
Anxiety	52	18.51
Depression	68	24.20
Bipolar disorder	16	5.69
Schizophrenia	6	2.14
Presenting symptom		
Nausea	403	98.29
Vomiting	403	98.29
Abdominal pain	341	83.17
Mean timing of HL (number of times given) ± SD	1.11 ± 0.33	
Median dose of HL (mg) ± IQR	2.5 ± 3.0	
Received 2nd doses	42	10.24
Received 3rd doses	2	0.49
HL was the first medication given	143	34.88
HL was the only medication given	52	12.68
Rout		
IV	347	84.63
IM	100	24.39
PO	5	1.22
EKGs performed before HL	151	36.83
EKGs performed after HL	105	25.61
Mean QTc (mm) ± SD before HL was given	450.43 ± 38.26	
Mean QTc (mm) ± SD after HL was given	458.87 ± 34.28	
Other medications given during the ED visit		
None	52	12.68
Opioids	142	34.63
Antiemetic medications	287	70.00
Benzodiazepines	53	12.93
H1-blockers, H2-blockers, and PPIs	168	40.98
ED total LOS (hr) ± SD	7.53 ± 3.91	
ED LOS before HL (hr) ± SD	3.16 ± 2.09	
ED LOS after HL (hr) ± SD	4.39 ± 3.41	
Side effects associated with HL		
None	392	95.61
Sedation	15	3.66
Dystonia	2	0.49
Akathisia	0	0.00
Cardiac arrhythmia	0	0.00
Disposition		
Home	232	56.59
Hospital admission	177	43.17
Transfer	0	0.00
Reason for admission to hospital		
Nausea	144	81.36
Vomiting	147	83.06
Abdominal pain	118	66.70
Other	52	29.38
Length of hospital stay (d) ± SD	3.49 ± 4.02	
Return to the ED within 30 d of discharge	136	33.17
Mean days of return to the ED ± SD	11.48 ± 8.97	
Was there any outpatient follow-up?	79	19.27

BMI, body mass index; CHS, cannabinoid hyperemesis syndrome; CVS, cyclic vomiting syndrome; ED, emergency department; EKG, electrocardiogram; GERD, gastroesophageal reflux disease; GI, gastrointestinal; H1 and H2, histamine 1 and 2 receptors; HL, haloperidol; *ICD-10*, *International Classification of Diseases, Tenth Revision*; IM, intramuscularly; IQR, interquartile range; IV, intravenous; LOS, length of stay; PO, oral; PPIs, proton pump inhibitors; PUD, peptic ulcer disease; QTc, corrected QT interval.

Of these 281 patients, 39.8% had diagnosis of gastroparesis, 28.3% had CHS, 10.5% had CVS, 5.8% had peptic ulcer disease, 3.7% had chronic abdominal pain, and 1.7% had gastroesophageal reflux disease (GERD). Anxiety was present in 18.5%, depression in 24.2%, bipolar in 5.7%, and schizophrenia in 2.1%.

Most common symptoms were nausea and/or vomiting (98.3%), and 83.2% of patients had abdominal pain. Most common *International Classification of Diseases, Tenth Revision* diagnostic codes were nausea with vomiting (31.5%), gastroparesis (17.1%), cyclic vomiting (9.8%), unspecified vomiting (7.8%), and intractable cyclic vomiting (5.1%) (Table 2).

**Table 2.** Correlation of demographic and other factors with outcomes

	ED LOS		Hospital LOS		How many days later did the patients return?	
	r	*P*	r	*P*	r	*P*
Age	**+0.13**	**0.007**	+0.08	0.277	**+0.17**	**0.041**
BMI	−0.02	0.672	+0.05	0.560	−0.04	0.639
Frequency of HL doses given	**+0.13**	**0.012**	−0.03	0.666	+0.07	0.412
HL dose	−0.01	0.832	−0.03	0.738	+0.10	0.231
ED LOS	—	—	+0.01	0.892	**+0.23**	**0.007**

The bivariate Spearman rho test was used for correlation between continuous variables. *P* is considered statistically significant at <0.05 (bolded).

BMI, body mass index; ED, emergency department; HL, haloperidol; LOS, length of stay.

These patients on average received HL 1.11 ± 0.33 times (range 1–3 times) with median dose 2.5 ± 3.0 mg per dose (range 0.5–10 mg), mostly intravenously (84.6%), followed by 24.4% intramuscularly, and 1.2% (5 patients) who received oral doses (Table 1). Approximately 10.2% received a second dose of HL, and 0.5% of patients received a third dose of the medication during their ED visit. HL was the first medication given in 34.9% of encounters, and it was the only medication given during the ED visit in 12.7% (52 patients). In addition to HL, 69.9% of patients also received antiemetic medications including 5-HT_3_ receptor antagonists (e.g., ondansetron) and dopamine receptor antagonists (e.g., metoclopramide), 34.7% of patients received opioids, 12% received benzodiazepines, and 17.8% were given acid-suppressive medications such as H1-, H2-blockers, or proton pump inhibitors.

Side effects related to HL occurred in 4.1% cases. The most common side effect was sedation in 3.7% of patients, followed by dystonia in 0.5% (2 patients). In patients who had electrocardiogram performed before and after HL administration, there was a trend toward longer QT interval (corrected QT interval before: HL: 450.4 ± 38.3 milliseconds, corrected QT interval after HL: 458.9 ± 34.3 milliseconds; *P* = 0.054), but no incidents of Torsade de Pointes were reported. No cases of severe adverse reactions including anaphylaxis or death were reported.

Total ED length of stay (LOS) was 7.5 ± 3.9 hours (3.1 ± 2.1 hours before HL administration and 4.4 ± 3.4 hours after). The majority of patients (56.6%) were discharged home after HL treatment while 43.2% were admitted to the hospital mostly because of persistent nausea and vomiting (62.1%) and abdominal pain (28.8%). Of patients admitted, the hospital LOS was on average 3.5 ± 4 days. Of people who were discharged from the ED, 136 patients (33.2%) returned to the ED within 30 days on average after 11.5 ± 9 days. Only 79 patients (19.3%) had any outpatient follow-up in the next 90 days after discharge.

Older patients had longer ED LOS (r = +0.13, *P* ≤ 0.01) and returned to the ED within 30 days, presented later (r = +0.17, *P* = 0.041) (Table 2). More frequent doses of HL also showed slight correlation with ED LOS (r = +0.13, *P* < 0.05). Patients who stayed in the ED longer during their initial visit and then returned within 30 days tended to present later (r = +0.23, *P* < 0.01).

When adjusted for other variables including age, sex, race, BMI, diabetes, underlying GI disorders, psychiatric disorders, presenting symptoms, and other medications given in the ED, patients with diabetes, cannabinoid use, anxiety, and those who received antiemetics in addition to HL, and those who stayed in the ED longer were more likely to be admitted to the hospital (odds ratio [OR] = 4.56, OR = 2.31, OR = 2.56, OR = 2.11, OR = 1.34, respectively, all *P* < 0.05) (Table 3). Patients who received HL as the only medication during their ED visit as well as patients who mainly presented with abdominal pain were less likely to be admitted (OR = 0.25, OR = 0.47, respectively, all *P* < 0.05). Patients who also received benzodiazepines in addition to HL were more likely to return to the ED within 30 days (OR = 2.28, *P* = 0.01), and African American patients, patients who used cannabinoids, were less likely to have outpatient follow-up within 90 days (OR = 0.36, OR = 0.47, respectively, *P* < 0.05). Admission to hospital led to higher rate of outpatient follow-up in this group (OR = 4.49, *P* < 0.01). HL dose received in the ED did not predict admission to the hospital, return to the ED within 30 days, or follow-up rate.

**Table 3.** Factors associated with outcomes in patients presenting with GI symptoms who receive HL

	Admission to hospital			Return to the ED within 30 d			Outpatient follow-up		
	OR	95% CI	*P*	OR	95% CI	*P*	OR	95% CI	*P*
Age	**1.05**	1.02–1.07	**0.000**	0.99	0.97–1.01	0.259	1.01	0.99–1.04	0.381
Sex (female)	1.00	0.55–1.81	0.998	0.67	0.41–1.1	0.117	1.72	0.87–3.4	0.119
BMI	1.01	0.97–1.05	0.565	1.01	0.97–1.04	0.799	1.01	0.97–1.05	0.740
Diabetes	4.56	2.06–10.1	0.000	1.50	0.77–2.93	0.237	0.91	0.4–2.06	0.822
Cannabinoid use	2.31	1.15–4.65	0.019	0.97	0.55–1.72	0.909	0.47	0.24–0.92	0.027
Chronic opioid use	0.59	0.29–1.17	0.129	0.81	0.46–1.43	0.459	1.27	0.62–2.59	0.510
Race									
African American	0.45	0.16–1.26	0.127	1.52	0.63–3.69	0.355	0.36	0.14–0.97	0.043
White	1.47	0.45–4.87	0.526	0.72	0.25–2.06	0.540	0.38	0.12–1.18	0.095
Latino	0.63	0.2–1.98	0.426	1.11	0.42–2.91	0.831	0.69	0.24–2	0.497
Psychiatric disorders									
Anxiety	2.56	1.09–6.01	0.031	1.26	0.61–2.59	0.534	1.63	0.71–3.75	0.255
Depression	0.87	0.43–1.77	0.695	1.20	0.65–2.2	0.561	1.15	0.55–2.4	0.705
Bipolar	1.78	0.53–5.93	0.350	1.04	0.38–2.84	0.935	1.43	0.46–4.45	0.537
Schizophrenia	4.02	0.83–19.52	0.085	1.94	0.51–7.37	0.332	0.45	0.08–2.5	0.364
Underlying GI disorders									
Gastroparesis	0.82	0.39–1.72	0.602	1.54	0.83–2.87	0.175	1.63	0.76–3.46	0.208
GERD	1.60	0.27–9.59	0.609	0.88	0.15–5.22	0.884	0.90	0.11–7.59	0.926
CVS	0.57	0.2–1.62	0.288	1.19	0.53–2.64	0.679	1.79	0.63–5.09	0.273
CHS	1.20	0.58–2.5	0.619	0.95	0.51–1.76	0.872	0.41	0.16–1.06	0.066
Main presenting chief complaint									
Abdominal pain	0.47	0.22–0.99	0.046	1.53	0.8–2.92	0.202	2.49	1.07–5.8	0.035
Nausea	0.08	0–3.5	0.189	1.94	0.2–18.9	0.569	2.32	0.2–26.48	0.499
Vomiting	0.13	0.01–2.84	0.194	0.22	0.03–1.71	0.146	0.25	0.02–2.48	0.234
Medications received during the ED visit									
No other medications other than HL	0.25	0.07–0.89	0.033	1.17	0.44–3.13	0.751	1.05	0.31–3.58	0.936
PPI, H1-, or H2-blockers	1.03	0.58–1.83	0.924	1.39	0.85–2.27	0.187	0.91	0.48–1.72	0.768
Antiemetics	2.11	1.03–4.32	0.042	1.62	0.86–3.06	0.135	0.90	0.41–2.01	0.803
Benzodiazepine	0.67	0.29–1.52	0.336	2.28	1.19–4.38	0.013	0.68	0.28–1.62	0.380
Narcotics	1.25	0.69–2.25	0.463	1.19	0.72–1.97	0.487	0.52	0.27–1.02	0.057
HL dose	1.05	0.88–1.25	0.593	1.07	0.93–1.24	0.331	0.95	0.79–1.14	0.577
Hospital parameters									
ED LOS	1.34	1.22–1.47	0.000	1.01	0.94–1.08	0.776	0.96	0.88–1.04	0.288
Admission				1.12	0.64–1.96	0.692	4.49	2.16–9.34	0.000
Outpatient follow-up				0.84	0.46–1.54	0.573			

Multiple binary logistic regression models were used to predict OR for categorical outcomes. *P* is considered statistically significant at <0.05 (bold).

BMI, body mass index; CHS, cannabinoid hyperemesis syndrome; CI, confidence interval; CVS, cyclic vomiting syndrome; ED, emergency department; GERD, gastroesophageal reflux disease; GI, gastrointestinal; H1 and H2, histamine 1 and 2 receptors; HL, haloperidol; LOS, length of stay; OR, odds ratio; PPI, proton pump inhibitor.

An additional analysis was performed comparing patients receiving HL for GI symptoms compared with a control group of patients who presented to the ED with GI symptoms but did not receive HL. This control group consisted of a total of 561 patients with 820 ED visits; average age 38.6 ± 14.2 years, 66.6% women, and BMI 27.9 ± 7.1. The same outcome variables (hospital admission, return to the ED within 30 days, and outpatient follow-up) were examined using multivariable regression models (Table 4). After adjusting for age, sex, BMI, opioid use, cannabinoid use, and comorbidities such as diabetes, gastroparesis, GERD, CVS, and CHS, patients who received HL as the only medication during their ED visit were less likely to be admitted to the hospital (OR = 0.25, *P* < 0.01). In the same model, DM, CHS, and gastroparesis were associated with higher hospital admission rate (OR = 2.13, OR = 1.72, OR = 2.75, respectively, all *P* < 0.05).

**Table 4.** Outcomes of all patients (who did and did not receive HL) presenting with gastrointestinal symptoms to the ED

	Admission to hospital			Return to the ED within 30 d			Outpatient follow-up		
	OR	95% CI	*P*	OR	95% CI	*P*	OR	95% CI	*P*
Received only HL in the ED	**0.25**	0.14–0.470	**0.000**	0.88	0.58–1.36	0.566	0.64	0.37–1.13	0.119
Age	**1.03**	1.02–1.05	**0.000**	1.00	0.99–1.02	0.897	**1.03**	1.02–1.05	**0.000**
Sex (female)	0.96	0.67–1.38	0.807	0.77	0.56–1.070	0.121	**1.67**	1.1–2.55	**0.017**
Diabetes	**2.13**	1.38–3.3	**0.001**	**1.54**	1.01–2.340	**0.046**	1.09	0.68–1.76	0.732
BMI	0.99	0.97–1.02	0.581	1.00	0.99–1.03	0.781	1.02	1–1.05	0.204
Chronic opioid user	1.31	0.87–1.97	0.198	0.89	0.61–1.31	0.548	**1.89**	1.23–2.91	**0.004**
Cannabinoid user	1.65	1.110–2.47	0.013	1.29	0.910–1.83	0.154	0.73	0.48–1.12	0.145
Cyclic vomiting syndrome	1.41	0.71–2.85	0.332	1.28	0.71–2.33	0.422	1.15	0.500–2.630	0.747
Cannabinoid hyperemesis syndrome	**1.72**	1.04–2.860	**0.037**	0.90	0.57–1.45	0.673	0.66	0.33–1.34	0.249
Gastroparesis	**2.75**	1.79–4.24	**0.000**	1.49	0.990–2.26	0.056	1.60	1–2.57	0.051
Gastroesophageal reflux disease	1.20	0.65–2.21	0.566	1.23	0.71–2.120	0.469	**3.17**	1.8–5.61	**0.000**

Multiple binary logistic regression models including all variables above were used to predict OR for categorical outcomes. *P* is considered statistically significant at <0.05 (bold).

BMI, body mass index; CI, confidence interval; ED, emergency department; HL, haloperidol; OR odds ratio.

## DISCUSSION

This study characterized the use and clinical outcomes of treating patients with HL for GI symptoms in the ED. Patients received HL primarily for treatment of nausea, vomiting, and abdominal pain. HL was mostly administered intravenously, usually as a single dose. Side effects to treatment (sedation and dystonia) occurred in only 4.4% of patients, and no serious side effects were reported. The 2 patients who had dystonic reactions received intravenous diphenhydramine with improvement, and they were discharged home. Of patients receiving HL, the majority (57%) were successfully treated and were able to be discharged from the ED.

The use of medications (both HL and other medications) influenced the ED discharge rate, and they were associated to one another. Patients who received antiemetics in addition to HL were more likely to be admitted, those who received benzodiazepine in addition to HL returned to the ED more often, and patients who received opioids in addition to HL were less likely to pursue outpatient follow-up. Interestingly, patients who received HL as the only medication during their ED visit were less likely to be admitted to the hospital (OR = 0.25, *P* < 0.05), and this was furthermore demonstrated in multivariable models of both groups (patients who received HL and those who did not). This may represent patients with less severe symptoms at presentation and less likely chance of being admitted a priori.

There is a paucity of research data for HL use for GI symptoms. In a similar fashion to our study, the Haloperidol Undermining Gastroparesis Symptoms trial used an EMR to examine 52 patients who presented to the ED with diabetic gastroparesis-related symptoms including nausea, vomiting, and abdominal pain and demonstrated a significant reduction in dose of opioid use and hospital admissions (14). Roldan et al. (15) in a double-blind, placebo-controlled trial randomized patients with gastroparesis in the ED to receiving HL or placebo and noted improvement in nausea and pain scores after 1 hour compared with the placebo group. Witsil and Mycyk (16) reported 4 cases of CVS patients who failed standard ED therapy improved significantly after receiving HL. As mentioned above, patients in our study who only received HL as the only medication in the ED were less likely to be admitted. Whether this is due to the efficacy of HL or patients receiving only HL had less severe symptoms, as repeat treatments were not needed, can be addressed in a prospective study of HL in the ED.

In this study, HL was primarily used in the ED for the GI symptoms of nausea, vomiting, and/or abdominal pain, often if patients had gastroparesis, CVS, use of cannabinoids, and/or opiates. HL was used off-label as the initial therapy for symptoms of nausea, vomiting, and/or abdominal pain in 35% of patients and was the only therapy used in 12.7% of patients. We do not know why HL was used first as there are a number of approved agents for the treatment of nausea, vomiting, and abdominal pain that could have been tried first. Patients with particularly severe symptoms seemed to be more likely to be treated with HL.

Our study evaluating the use of HL in the ED has several design advantages. The study looked at a defined period, searching for anyone receiving HL. Our ED is a tertiary care center, seeing a variety of patients including a number of minority populations. Our study, however, did have several limitations. As a retrospective chart review-based study, we were mostly relying on the information available in the electronic medication records. We were not able to assess the severity of symptoms and how successfully their symptoms improved. We used successful discharge from the ED as a favorable outcome variable to treatment with HL. Although the EMR systems and several medical centers are integrated and available, some information such as outpatient follow-ups by physicians outside our network could be missing. This study examined patients presenting to a single tertiary care center ED in a busy urban area which may translate into sicker patients with more complex medical and social issues. Furthermore, as neither patients nor physicians were blinded to receiving HL and as there are no specific guidelines for ED physicians to use HL for GI symptoms, there is a potential for selection bias, that is physicians gave HL more frequently for cannabinoid or opiate users.

In conclusion, HL is being used in the ED to treat patients with GI symptoms, particularly nausea, vomiting, and abdominal pain in patients with a variety of disorders including gastroparesis, CVS, CHS, and other disorders. Our study shows that HL has promising results for GI symptoms in the ED including reducing the admission rate to the hospital and was safe with low rates of adverse events. If larger prospective, placebo-controlled studies demonstrate the efficacy and safety of HL for GI symptoms, these results could potentially support using HL sooner and more broadly which may lead to fewer hospital admissions, shorter ED visits, and less frequent return to the ED. Future directions of study can also include the continued use of HL as an outpatient after ED discharge.

## CONFLICTS OF INTEREST

**Guarantor of the article:** Dariush Shahsavari, MD.

**Specific author contributions:** D.S.: study planning, data collection and analysis, literature review, and writing the manuscript. K.R-L.: data collection and literature review. Z.M., M.W., A.J., and Z.D.R.: study planning, writing and critical revision of the manuscript for important intellectual content. H.P.P.: study planning and supervision of the study, data analysis, literature review and writing, and critical revision of the manuscript.

**Financial support:** None to report.

**Potential competing interests:** None to report.

**Study Highlights**

**WHAT IS KNOWN**

✓ Gastrointestinal (GI) symptoms including nausea, vomiting, and abdominal pain are responsible for a significant number of visits to emergency departments (EDs).
✓ Haloperidol (HL), a first-generation antipsychotic agent, has been successfully used for nausea and vomiting in surgical and cancer patients.
✓ HL also has shown efficacy in cyclic vomiting syndrome, cannabinoid hyperemesis syndrome, and gastroparesis.
✓ In the absence of clear clinical guidelines, current trends of HL use and its usefulness for GI symptoms in the ED are poorly characterized.

**WHAT IS NEW HERE**

✓ Most patients improved and were discharged home after receiving HL.
✓ Patients who received HL as the only medication during their visit were less likely to be admitted to the hospital.
✓ Older age, diabetes, cannabinoid use, anxiety, having abdominal pain, receiving antiemetics, and spending more time in the ED was associated with higher hospital admission.
✓ Cannabinoid users and African Americans were less likely to have outpatient follow-ups.

**TRANSLATIONAL IMPACT**

✓ Haloperidol seems to be an effective and relatively safe treatment option for GI symptoms including nausea, vomiting, and abdominal pain, and shows promising results in patients presenting to the emergency department including reduction in hospital admissions.
